# Effect of an Acupuncture Technique of Penetrating through *Zhibian* (BL54) to *Shuidao* (ST28) with Long Needle for Pain Relief in Patients with Primary Dysmenorrhea: A Randomized Controlled Trial

**DOI:** 10.1155/2019/7978180

**Published:** 2019-12-17

**Authors:** Haijun Wang, Yuxia Cao, Xiaofei Jin, Min Yan, Jianchao Wang, Rangqian Li, Laixi Ji

**Affiliations:** ^**1**^ Shanxi University of Traditional Chinese Medicine, Jinzhong, Shanxi, China; ^2^The Third Teaching Hospital of Shanxi University of Traditional Chinese Medicine, Taiyuan, Shanxi, China

## Abstract

**Background:**

Primary dysmenorrhea (PD) is the commonest gynecological disorder in young women of reproductive age, and there is not always satisfactory relief of pain treated by common medications. Therefore, acupuncture has been used as an alternative therapy to relieve the symptoms of PD. In clinical practice, a penetrating method of acupuncture with long needle has been shown to be particularly effective for improving primary dysmenorrhea. This study was conducted to evaluate the effectiveness of this technique for pain relief in patients with primary dysmenorrhea as compared with a conventional pain medication.

**Methods:**

The present study is a perspective, randomized, ibuprofen-controlled trial. Sixty-two eligible participants were randomly assigned in a 1 : 1 ratio to receive either acupuncture treatment or ibuprofen administration. The treatment lasted for three menstrual cycles for both groups. The primary outcome was the intensity of menstrual pain measured by using the visual analogue scale at the completion of treatment. Secondary outcomes included the severity of symptoms associated with menstrual pain, responder rate, and safety of acupuncture treatment. The clinical outcomes were measured on each menstrual cycle at baseline, treatment course (3 cycles), and follow-up period.

**Results:**

Sixty-four patients of primary dysmenorrhea were recruited, and 62 subjects were included in the final analysis. At trial completion, acupuncture was shown to be associated with a significantly lower pain intensity and decreased symptom severity of primary dysmenorrhea as compared with ibuprofen (*p* < 0.05). A significantly higher responder rate was found in the acupuncture group as compared with the control group (*p* < 0.05). No serious adverse events were reported by patients in either group.

**Conclusions:**

The penetrating method of acupuncture with long needle may be an effective and safe therapy for pain relief in patients with primary dysmenorrhea. This trial was registered with the Chinese Clinical Trial Registry (ChiCTR-IOR-17012621).

## 1. Background

Primary dysmenorrhea is a main gynecological disorder in the absence of significant pathological changes which prevails in adolescent girls in the first 2-3 years after menarche or in young women who have not given birth [1]. The prevalence of primary dysmenorrhea in menstruating women is ranged between 45% and 72% [2,3]. Nonsteroidal anti-inflammatory drugs (NSAIDs) are the first recommendation to temporarily relieve pain, which are often ineffective or slow-acting [4], and at the same time adverse effects are reported such as indigestion, headache, and drowsiness, [5]. Consequently, the alternative therapies, especially acupuncture, have been used as the important treatment [6].

Recently, there is a growing interes in using acupuncture to relieve menstrual pain [7–10]. Many clinical studies showed that acupuncture can not only help reduce pain intensity and improve pain coping but also diminish menstrual pain associated symptoms such as emotional distress and reduced quality of life [11]. A recent systematic review found that there was insufficient evidence to demonstrate whether acupuncture provides additional benefits of pain relief to patients with menstrual pain as compared with pain medications, as a result of substantial heterogeneity [12]. To our knowledge, one major reason is due to the variety of the “dose” of acupuncture such as selected acupoints and thus the lack of treatment standardization [13]. We believe the choice of standardized yet optimally effective acupoints is vital to induce an analgesic effect by acupuncture for primary dysmenorrhea. In our clinical practice, we noticed that the technique of acupuncture penetrating through Zhibian (BL54) to Shuidao (ST28) with long needle is associated with satisfactory effect in treating patients with primary dysmenorrhea. Based on such clinical observations, we hypothesized that this acupuncture technique with long needle penetration would reduce menstrual pain severity and relieve other symptoms after treatments of 3 cycles and, therefore, conducted this randomized controlled trial to assess the clinical effect by comparing with conventional pain medication.

## 2. Materials and Methods

### 2.1. Study Design

This study utilized a single-centered, randomized, ibuprofen-controlled trial, and the ratio of patients in the treatment group to control group was 1 : 1. The consolidated Standards of Reporting Trial and the Revised Standards for Reporting Interventions in Clinical of Acupuncture in designing this study were followed [14]: this study was reviewed by the Ethics Review Committee of the 3^rd^ Teaching Hospital of Shanxi University of Traditional Chinese Medicine on Dec 12, 2013 (Approval NO. 2013A-028), and registered at the Chinese Clinical Trial Registry (registration no. ChiCTR-IOR-17012621) in 2017.

### 2.2. Participants

Patients with primary dysmenorrhea mainly were recruited from Shanxi University of Traditional Chinese Medicine by poster advertisement. All participants were explicitly explained about the study procedures and joined in this trial voluntarily by signing a written consent form, who have the right to participate or dropout at any time.

The following criteria were used in on-site screening to identify eligible participants:

- Inclusion criteria: (1) aged from 18 to 35 years without history of delivery; (2) normal menstrual cycle (28 ± 7 days) in previous 3 months; (3) diagnosed with PD according to the Primary Dysmenorrhea Consensus Guidelines and confirmed no significant organ pathologies with ultrasound B [15]and with different pattern based on the revised Chinese National Guideline [16]; (4) moderate to severe menstrual pain intensity with a score of >40 mm on the Visual Analog Scale (VAS); and (5) able to understand the whole study and agree with all procedures by signing a written informed consent
- Exclusion criteria: (1) secondary dysmenorrhea due to other gynecological diseases confirmed by an obstetrician visit or gynecological abdominal ultrasound B examination; (2) serious diseases such as progressive central nervous disorders, AIDS, serious infection, psychiatric disorders, malignant tumor, tuberculosis, hematological diseases, diabetes mellitus, and damaged hepatic/renal function; (3) receiving or having received any other treatment for PD in previous two weeks; (4) severe optoacoustic or cognitive dysfunction; (5) cutaneous infection or spontaneous bleeding disorder; and (6) pregnant or lactating women

### 2.3. Sample Size Estimation

Sample size was calculated by using G *∗* Power 3 software (Institute for experimental psychology, Heinrich Heine university, German). For this trial, it has been determined prospectively that *α* = 0.05 and 1 − *β* = 0.90. According to a previous trial on acupuncture for PD [17], a total of 64 participants will be included in this trial for a compensation to 15% dropout rate, with 32 patients in each group.

### 2.4. Randomization and Blinding

A random sequence was generated using the random digit table and sealed in opaque envelopes by an independent researcher who was blinded to the group allocation. A research assistant who handled patient recruitment unsealed one of the envelopes and allocated the participants to each designated group. As an open-label trial, neither the acupuncturists who delivered the acupuncture treatment nor the physician who prescribed ibuprofen was blinded to the group assignment. The clinical outcomes were initially assessed by a third researcher who was blinded to the ground assignment and further analyzed by an independent statistician.

### 2.5. Interventions

#### 2.5.1. Acupuncture Treatment Group

A semistandardized acupuncture treatment scheme was applied, which included manual stimulation at fixed acupoints including Zhibian (BL54) and Shuidao (ST28) and individualized acupoints. For a patient with a TCM diagnosis of stagnation of coldness and dampness, moxibustion would be applied at Shuidao (ST28); for the pattern of Qi stagnation and blood stasis, Hegu (LI4), Taichong (LR 3), and Ciliao (BL32) would be stimulated with manual acupuncture; for the dual deficiency of Qi and blood, Xuehai (SP10), Pishu (BL20), and Zusanli (ST36) would be manually stimulated.

**Figure 1:**
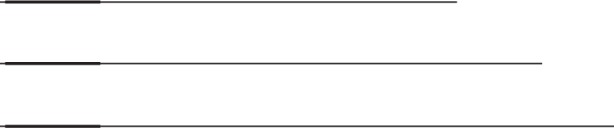
The long needles.

**Figure 2:**
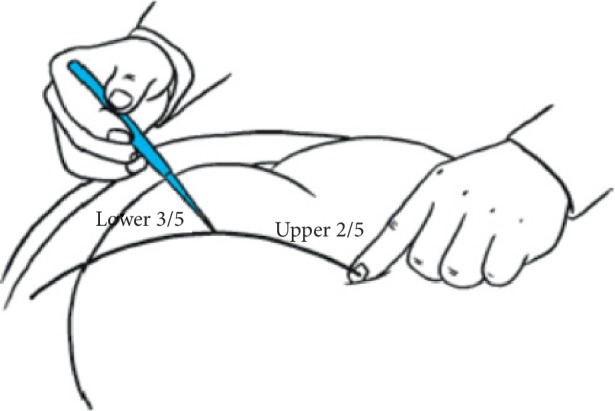
The acupuncture spot of penetrating through *Zhibian* (BL54) to *Shuidao* (ST28).

**Figure 3:**
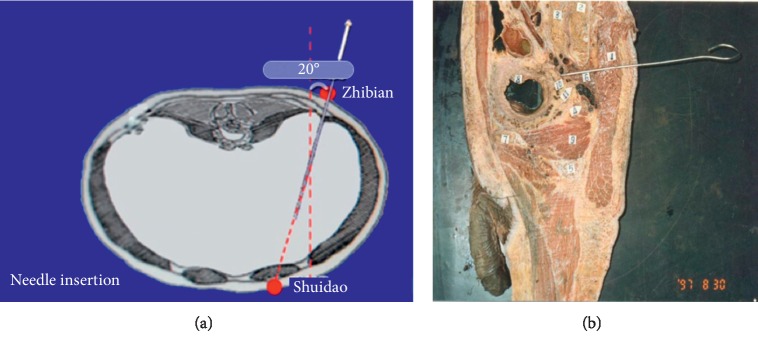
The direction and the angle of penetrating through *Zhibian* (BL54) to *Shuidao* (ST28).

During acupuncture treatment, patients were asked to lie in a prone position. An acupuncturist would initially locate the acupoints to be stimulated. After skin disinfection with a 75% alcohol swab, a long needle (length: 5–7 cun/12.5–17.5 cm, diameter: 0.32 mm) (Figure 1) was inserted from Zhibian (BL54) with its tip pointing to Shuidao (ST28) (Figures 2 and 3). The needle was inserted obliquely at the convergent point located on upper two-fifths and lower three-fifths of the line which was between the inner edge of the posterior superior iliac spine and the inner edge of the greater trochanter of the femur, to form an angle of 20° with the sagittal axis of patient's body(Figure 2). During the insertion, an acupuncturist used her thumb and index finger of the right hand to guide the needle body with supports from right-hand fingers holding the rest of the needle body. The depth of insertion was 4 to 6 cun, and Deqi sensation in the pubic area or the lower abdomen was achieved. The needle was further twirled and rotated for 1 min and then retained at acupoints for 20 min (in interval needle manipulation). For other points, the routine needling technique was applied, and needles were left in place for 30 min. Acupuncture treatment was commenced at the fifth day prior to the anticipated menstruation and repeated once daily till the onset of menstruation. The treatment lasted for three menstrual cycles. During acupuncture treatment, other prophylactic drugs were prohibited; however, emergent use of painkillers was allowed. The time, dose, and pain relief after drug use were required to be explicitly recorded in the menstrual diary. In order to ensure the homogeneity of acupuncture treatment, all sessions were delivered by one certified acupuncturist with over 5 years of clinical experience in the outpatient setting of the Department of Acupuncture in the 3^rd^ Teaching Hospital.

#### 2.5.2. Ibuprofen Group

Patients in the medication group were instructed to administer ibuprofen sustained-release capsule orally (Sino-US Tianjin Shike Pharmaceutical Co., Ltd., national medicine standard ID: H10900089, specification: 300 mg/granule), twice daily (once every 12 hours) starting at five days prior to the onset of menstruation till the first day of menstruation. Medications were used for 3 menstrual cycles.

### 2.6. Outcomes

The primary outcome was intensity of menstrual pain measured by using the Visual Analogue Scale (VAS) at the completion of treatment [18]. Secondary outcomes included symptom severity of menstrual pain, treatment response rate defined as the proportion of patients with at least 25% reduction of symptom severity score after treatment, and safety measured by the incidence of adverse events. Pain intensity and symptom severity were measured at baseline (1 cycle) during treatment course (3 menstrual cycles) and after treatment completion. Patients were required to keep menstrual dairies to track their menstrual pain conditions during the whole study period.

### 2.7. Statistical Analysis

The full analysis set (FAS) of study data was analyzed using the intention-to-treat (ITT) strategy. Missing data was imputed by carrying forward the results of the last observation. Statistical analysis was completed using SPSS 18.0 (IBM Corporation, Chicago, the U.S.). For continuous data, the independent sample *t*-test was used under the assumption of normal distribution. Otherwise, nonparametric tests such as the chi-square test would be used. For dichotomous data, the Wilcoxon rank sum test and chi-square test were used. A two-sided *p* value < 0.05 indicates a statistically significant difference.

## 3. Results

**Figure 4:**
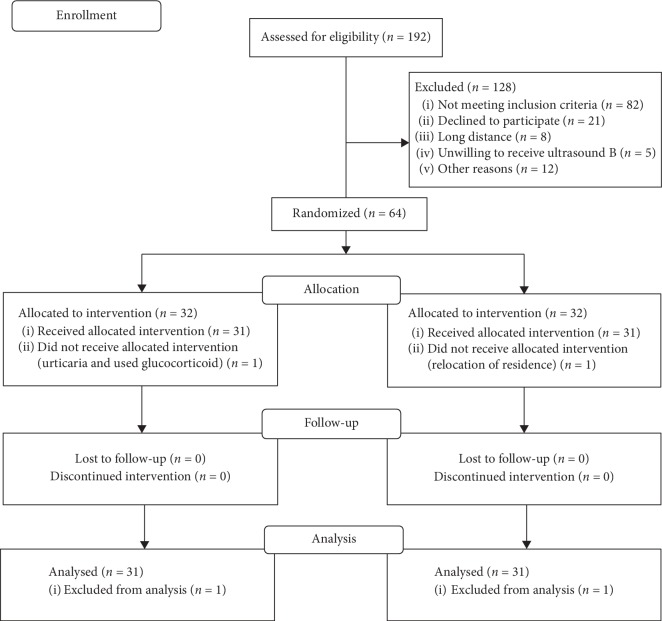
Study flowchart.

There were 64 patients with primary dysmenorrhea recruited from the 3^rd^ Teaching Hospital of Shanxi University of Traditional Chinese Medicine from May 2016 to August 2017. Before initiation of the treatment course, one patient withdrew from the acupuncture group due to developing urticaria and intaking glucocorticoid; one patient in the medication group withdrew due to graduation and relocation of residence. Finally, due to availability of baseline data, only 62 subjects were included in the ITT analysis (Figure 4).

### 3.1. Clinical Characteristics and Demographics of Included Participants

The demographic information showed that two groups were comparable in terms of patient age and disease course. Moreover, patients in two groups were comparable in terms of baseline menstrual pain intensity and symptom severity. The stratifications of patients based on severity levels were well balanced across two groups (Table 1).

### 3.2. Pain Relief and Symptom Improvement after Treatment

The intensity of menstrual pain at the completion of treatment was significantly lower in the acupuncture group than that in the ibuprofen group (*p* < 0.0001). Compared with baseline, both groups were associated with significant pain reduction (*p* < 0.05). Moreover, after treatment, the symptom severity of menstrual pain in the acupuncture group was significantly lower than that in the ibuprofen group (*p* < 0.0001). There was also a significant change of symptom severity at the post-treatment visit as compared with baseline in both groups (*p* < 0.05). Further analysis showed that the response rate of the acupuncture group was significantly higher than that of the ibuprofen group (*p* < 0.05) (Table 2).

### 3.3. Adverse Events

During the whole trial period, patients in the acupuncture group reported 7 adverse events including subcutaneous hemorrhage occurred during or after treatment. Such symptoms resolved properly within 7 days after compression with hot towel. In the ibuprofen group, 17 patients developed gastrointestinal irritation, such as nausea, anorexia, heartburn, and bloating. The side effects of drugs resolved properly after the cessation of drug use.

## 4. Discussion

This randomized drug-controlled trial showed that, after three cycles of acupuncture treatment with specific techniques, known as the penetration through Zhibian (BL54) to Shuidao (ST28) with long needle, menstrual pain intensity was significantly reduced and menstrual pain-related symptoms were improved in patients with primary dysmenorrhea. Moreover, acupuncture was associated with a higher response rate and low incidence of adverse events, as compared with a conventional analgesic, ibuprofen.

Previous studies mainly compared acupuncture with a sham control and found that acupuncture is effective in relieving pain and easing emotional distress in dysmenorrheal patients [19]. Based on clinical evidence, the Society of Obstetrician and Gynecologist of Canada stated in the clinical guideline that acupuncture was superior to placebo and to Chinese herbs in the relief of menstrual pain [20]. However, there are still controversies regarding the benefits of acupuncture for females with menstrual pain, according to a recent Cochrane systematic review [18]. Possible reasons leading to the discrepancy may include treatment timing, mode of needle stimulation, needle location, number of needles used, and frequency of treatment, all of which are deemed vital to the achievement of an optimal acupuncture effect [21]. At present, a pragmatic trial that compares acupuncture with conventional pain medications is still insufficient. Our pragmatic randomized trial showed that acupuncture was superior to ibuprofen in reducing menstrual pain intensity and alleviating other symptoms. The results are consistent with our clinical observations and the aforementioned studies.

In traditional Chinese medicine theory, dysmenorrhea is caused by unsmoothing flowing of Qi and blood. Acupuncture has been widely used to treat pain condition and an essential aspect of its mechanism is to promote Qi and blood circulation in meridians [22]. The specific needling technique being studied in this study is the penetration of acupoint from Zhibian (BL54) to Shuidao (ST28) with a long needle. This technique is based on not only traditional Chinese medicine theories but also knowledge of local tissue anatomy and a number of autopsy experience. The author has conducted an in-depth exploration in the angle, direction, and safety of the penetrating method from Zhibian(BL54) to Shuidao(ST28), and clarified the operational feasibility and safety of such method. This technique is able to induce Qi arrival during which the patient may feel the radiation of needling sensation in the lower abdomen and the pubic or pelvic regions and even distention, warmth, and relief sensations in the pelvic cavity. According to our observation, the stronger the needle sensation is, the better the effect is. The possible mechanism is that the long needle may directly stimulate the sympathetic and parasympathetic nerve fibers in the pelvic plexus to further regulate their functions and reduce pain [23].

This study has several limitations. First of all, the sample size is small. Only 64 subjects were included in this trial. The observed effectiveness may represent a random effect and be caused by chance. Future adequately powdered pragmatic trials are warranted. Secondly, even though a pragmatic trial, this study has not included a placebo control and the detected effect of acupuncture may indicate an effect of nonspecific effect. Thirdly, the measurement of symptom severity has not underwent thorough scientific validation and thus may introduce uncertainty about the effect of acupuncture for menstrual pain severity. Moreover, the clinical response has been defined as the ration of subjects with at least 25% reduction of the VAS score, which is a cutoff point lower than the commonly used (30% in pain research). This study also does not have a long-term follow-up schedule, which makes it unable to identify the long-term effect of acupuncture. Future pragmatic trials with improvement in sample size, control selection, outcome measurement, and follow-up schedule are still needed.

## 5. Conclusion

This randomized, medication-controlled trial showed that 3-cycle treatment of a specific acupuncture technique of penetrating through Zhibian (BL54) to Shuidao (ST28) with a long needle was superior to ibuprofen in reducing menstrual pain intensity and improving symptom in patients with primary dysmenorrhea.

## Figures and Tables

**Table 1:** Clinical characteristics and demographics of included participants.

Group	Acupuncture (*n* = 31)	Ibuprofen (*n* = 31)
Age (yrs)	21.70 ± 4.08	21.90 ± 4.15
Disease course (yrs)	6.62 ± 1.53	6.55 ± 1.59
VAS score	7.62 ± 1.53	7.55 ± 1.59
Symptom severity score	13.78 ± 4.35	13.61 ± 4.15
Mild cases (*n*)	4	3
Moderate cases (*n*)	9	10
Severe cases (*n*)	18	18

**Table 2:** Change in clinical outcomes in both groups after treatment.

Group	Acupuncture (*n* = 31)	Ibuprofen (*n* = 31)	*p* value
VAS score	3.26 ± 1.49	5.71 ± 1.32	<0.0001
Symptom severity	3.32 ± 0.91	8.27 ± 3.08	<0.0001
Response rate^ (%)	100	87.1	<0.05
Adverse events (*n*)	7	17	—

^Note: response rate = (the number of subjects with at least 25% pain intensity reduction in one group)/(the total number of subjects in one group) *∗* 100%.

## Data Availability

The individual participant data (deidentified) used to support the findings of this study are available from the corresponding author upon request.

## Supplementary Materials

Supplementary Materials Checklist for items in STRICTA 2010 and CONSORT 2010 checklist of information to include when reporting a randomised trial.
